# Preparing for adulthood: thousands upon thousands of new cells are born in the hippocampus during puberty, and most survive with effortful learning

**DOI:** 10.3389/fnins.2014.00070

**Published:** 2014-04-23

**Authors:** Daniel M. Curlik, Gina DiFeo, Tracey J. Shors

**Affiliations:** Department of Psychology, Behavioral and Systems Neuroscience, Center for Collaborative Neuroscience, Rutgers UniversityPiscataway, NJ, USA

**Keywords:** neurogenesis, dentate gyrus, hippocampus, eyeblink conditioning, adolescence, pubescence

## Abstract

The dentate gyrus of the hippocampal formation generates new granule neurons throughout life. The number of neurons produced each day is inversely related to age, with thousands more produced during puberty than during adulthood, and many fewer produced during senescence. In adulthood, approximately half of these cells undergo apoptosis shortly after they are generated. Most of these cells can be rescued from death by effortful and successful learning experiences (Gould et al., 1999; Waddell and Shors, 2008; Curlik and Shors, 2011). Once rescued, the newly-generated cells differentiate into neurons, and remain in the hippocampus for at least several months (Leuner et al., 2004). Here, we report that many new hippocampal cells also undergo cell death during puberty. Because the juvenile brain is more plastic than during adulthood, and because many experiences are new, we hypothesized that a great number of cells would be rescued by learning during puberty. Indeed, adolescent rats that successfully acquired the trace eyeblink response retained thousands more cells than animals that were not trained, and those that failed to learn. Because the hippocampus generates thousands more cells during puberty than during adulthood, these results support the idea that the adolescent brain is especially responsive to learning. This enhanced response can have significant consequences for the functional integrity of the hippocampus. Such a massive increase in cell proliferation is likely an adaptive response as the young animal must emerge from the care of its mother to face the dangers, challenges, and opportunities of adulthood.

## Introduction

The brain continues to generate new neurons across the lifespan, through the process of neurogenesis. This process occurs in a wide variety of species, including humans (Altman and Das, 1965; Kaplan and Hinds, 1977; Eriksson et al., 1998). One region where this neurogenesis occurs is the dentate gyrus of the hippocampus, a structure required for many forms of learning and memory (Scoville and Milner, 1957; Weiss et al., 1999; Beylin et al., 2001). Each day, the adult dentate gyrus produces thousands of new cells (Gould et al., 1999; Cameron and McKay, 2001). Interestingly, most of these cells die within a few weeks of their birth. In adults, the majority of these cells could be rescued from death by new and effortful learning (Curlik and Shors, 2011; Shors et al., 2012; Curlik et al., 2013). However, there is a critical period during which learning keeps the cells alive; cells that are between 1 and 2 weeks of age at the time of training can be rescued from death by learning, whereas cells older or younger at the time of training do not necessarily survive (Epp et al., 2007; Anderson et al., 2011). The cells that are rescued by learning remain in the dentate gyrus for at least several months (Leuner et al., 2004), as they become integrated into the existing hippocampal circuit (Van Praag et al., 2002; Gu et al., 2012).

Thousands upon thousands of new cells can be generated each day, but the exact number varies with chronological age (Seki and Arai, 1995; Epp et al., 2009). Approximately 17,000 new cells are produced each day in dentate gyrus of peri-pubescent rats, whereas approximately 9,000 cells are produced each day in the dentate gyrus of young adult rats (Cameron and McKay, 2001). In general, the adolescent dentate gyrus produces 100–300% more cells than the adult dentate gyrus (Cameron and McKay, 2001; He and Crews, 2007; Hodes et al., 2009). In addition to age, the number of cells produced can vary greatly depending on a variety of environmental conditions, including housing, stress, and sex differences. However, it is difficult to estimate the exact numbers produced, because many of the cells die soon after they are generated. To our knowledge, there have been no studies that have quantified the number of new hippocampal cells that die during puberty. In the present study, we hypothesized that most of the new cells generated during puberty would die as they do during adulthood. We predicted that the time course of cell death in the adolescent dentate gyrus would mirror that of the adult dentate gyrus. Therefore, the majority of cells would die between the first and third week of their birth (Anderson et al., 2011). Because the adolescent brain is relatively plastic, we further hypothesized that a large proportion of the new cells would survive in pubescent animals that were trained to learn a task which is known to prevent cell death during adulthood. This task, trace eyeblink conditioning, is used to assess an animal's ability to associate one stimulus, in this case a white noise, with another stimulus, eyelid stimulation, across a temporal gap. Learning the association keeps new hippocampal cells alive, as long as the association is sufficiently difficult to acquire, and the animal learns well (Waddell and Shors, 2008; Curlik and Shors, 2011).

In the current studies, we adopted a peri-pubescent rat model of adolescent development (Ojeda and Urbanski, 1994), which spans the juvenile period, postnatal days 21 through 35 (PND21–35), through puberty (PND35–56). This model has been widely used to examine a variety of developmental changes, including changes to the process of hippocampal neurogenesis (Hodes et al., 2009). While juveniles, groups of rats were injected with BrdU to label one cohort of newly-generated cells. A subset of these animals were trained with trace conditioning one week after the BrdU injection, corresponding to the time course when the newly generated cells begin to undergo apoptosis during adulthood. The number of BrdU-positive cells remaining in the dentate gyrus of trained and untrained animals was assessed three weeks after labeling with BrdU, at a time point when the newly-generated cells would have differentiated into neurons in the adult brain.

## Methods

### Subjects

Male Sprague-Dawley rats were weaned from their mothers and singly housed at the beginning of the juvenile period, during postnatal days 21 through 23 (P21–23). All animals received one single-intraperitoneal injection of 5-bromo-2-deoxyuridine (200 mg/kg, BrdU) on PND26–27. BrdU is a thymidine analog, which incorporates into the DNA of cells that are currently in the S-phase of the cell cycle at the time of, and shortly after, administration. One intraperitoneal injection of a sufficient dose of BrdU will provide a “snapshot” of nearly all of the cells currently dividing in an animal at the time of the injection, including those in the dentate gyrus (Cameron and McKay, 2001). All rats were provided access to food and water *ad libitum*. Animals were maintained with a constant 12 h light/dark cycle. The light cycle began at 7 a.m. and ended at 7 p.m. All procedures were designed to fully comply with the PHS Policy on Humane Care and use of Laboratory Animals, and the Guide for the Care and use of Laboratory Animals. Rutgers University Animal Care and Facilities Committee approved the procedures.

### Experimental design

In the adult dentate gyrus, more than half of new cells undergo apoptosis between the first and third week of their birth (Gould et al., 1999; Anderson et al., 2011). In this study, we first determined whether cells generated in the adolescent dentate gyrus undergo cell death during this critical period. Two groups of experimentally naïve animals were administered BrdU during either PND26 or 27. Following BrdU-administration animals were returned to their home cage for the duration of the experiment. Seven days later, which corresponds to a time point when the cells have yet to die in adult rats, these animals were provided an overdose of pentobarbital (*N* = 6). The second group was treated similarly, but perfused three weeks after the BrdU injection, at a time point when many of the cells are no longer present in adults (*N* = 10). All cells in the dentate gyrus labeled with BrdU were counted and tallied for statistical analysis.

It has been reported that most of the newly-generated cells in the adult dentate gyrus that would have otherwise died instead survive in animals that participate in successful and effortful learning (Waddell and Shors, 2008; Anderson et al., 2011). To determine whether learning prevents cell death in the adolescent animal, we administered a single injection of BrdU to a new group of pubescent animals on PND26 or 27 (*N* = 9). One week after the injection, and just before the new cells begin to die, these animals were trained with four days of trace eyeblink conditioning. These trained animals were perfused three weeks after the BrdU injection, and the number of surviving BrdU+ cells in the dentate gyrus was counted and compared to that of untrained animals at the early (one week after the injection) and late time points (two weeks after the injection).

### Trace eyeblink conditioning

To assess learning, we trained animals with classical eyeblink conditioning, with a trace procedure. This procedure requires a short surgical operation. Prior to training, animals were anesthetized with isoflurane gas during PND21–23. Four stainless steel electrodes (0.005 inch diameter) were placed through the *orbicularis occuli*, and attached to a plastic headstage, which was secured to four small screws partially embedded within the skull (Servatius and Shors, 1996). Two electrodes were attached to the eyelid in order to deliver the unconditioned stimulus (US), which was an electrical pulse used to elicit an eyeblink, the unconditioned response (UR). The remaining two electrodes were used to record electromyographic activity of the muscle of the eyelid, which was used to detect the expression of a conditioned response (CR). All animals were provided at least 3 days to recover before receiving one single intraperitoneal injection of BrdU (PND26 or 27). As described above, groups of naïve animals were not trained, and were instead perfused either one or three weeks, after the BrdU injection. Naïve rats did not undergo surgery, and they remained in their home cage for the duration of the experiment. Like trained animals, naïve animals received one single injection of BrdU between PND26 and PND27.

Six days after the BrdU injection, and near the onset of pubescence (between PND33 and 34), trained animals were acclimated to the conditioning chambers for 1 h while spontaneous blinking activity was recorded. The next day (one week after the BrdU injection), all animals began training with trace eyeblink conditioning. Animals were trained with 200 trials of trace eyeblink conditioning per day, for four days. Each trial consisted of a 250 ms 82 dB white noise conditioned stimulus (CS), followed by a 500 ms stimulus free trace interval, which was followed by the US, 100 ms of 0.65 mA periorbital stimulation to the *orbicularis occuli*. The intertrial interval was 25 ± 5 s. The occurrence of an eyeblink was determined from EMG recordings of the muscle of the right eyelid. During each trial, baseline EMG activity was recorded 250 ms before presentation of the CS. An increase in EMG activity was considered a CR if the EMG activity during the trace interval was greater than the average amplitude of the baseline recording for that trial, plus four times the baseline recording's standard deviation. The ability to emit 60% CRs during any block of 100 trials was used as a measure of successful learning. This criterion has been previously used as a marker of successful learning, because the majority of adult animals successfully reach this criterion, and it strongly correlates with other similar learning criterion, such as the number of trials required to emit eight out of nine consecutive CRs (Curlik and Shors, 2011).

### Immunohistochemistry for BrdU

All animals were deeply anesthetized with sodium pentobarbital (100 mg/kg) and transcardially perfused with 4% paraformaldehyde. Brains were extracted and post-fixed in 4% paraformaldehyde at 4°C for 24 h before being transferred to phosphate buffered saline (PBS). Forty-micrometer coronal sections were cut with a Vibratome through the entire rostral-extent of the dentate gyrus of one hemisphere. Every twelfth section of the dentate gyrus was mounted to a glass slide and stained for the presence of BrdU with peroxidase methods. Sections were pretreated in 0.1 M citric acid, rinsed with PBS, and incubated in trypsin for 10 min. The tissue was denatured in 2N HCl for 30 min, rinsed with PBS, and incubated overnight in primary anti-mouse BrdU (1:200 Becton Dickson) and 0.5% Tween 20. The next day the tissue was rinsed with PBS and placed in anti-mouse secondary antibody for 60 min (1:200, Vector Laboratories). Sections were placed in avidin-biotin complex (1:100, Vector Laboratories) for 60 min, exposed to diaminobenzidine for four min and counterstained with cresyl violet. Slides were coverslipped with Permount glue (Fischer Scientific, Fair Lawn, NJ). The slides were coded and the experimenter was blind to the experimental condition of each slide. The number of BrdU+ cells in the dentate gyrus (granule cell layer + hilus) of each section was assessed using a light microscope (Nikon Eclipse 80i). The total number of BrdU+ cells from each animal was multiplied by 24 (2 hemispheres X every twelfth section) to estimate the total number of BrdU-positive cells in the entire dentate gyrus of each animal. Representative photomicrographs were taken with a DS-Fi1 Nikon camera using NIS Elements software.

## Results

**Figure 1:**
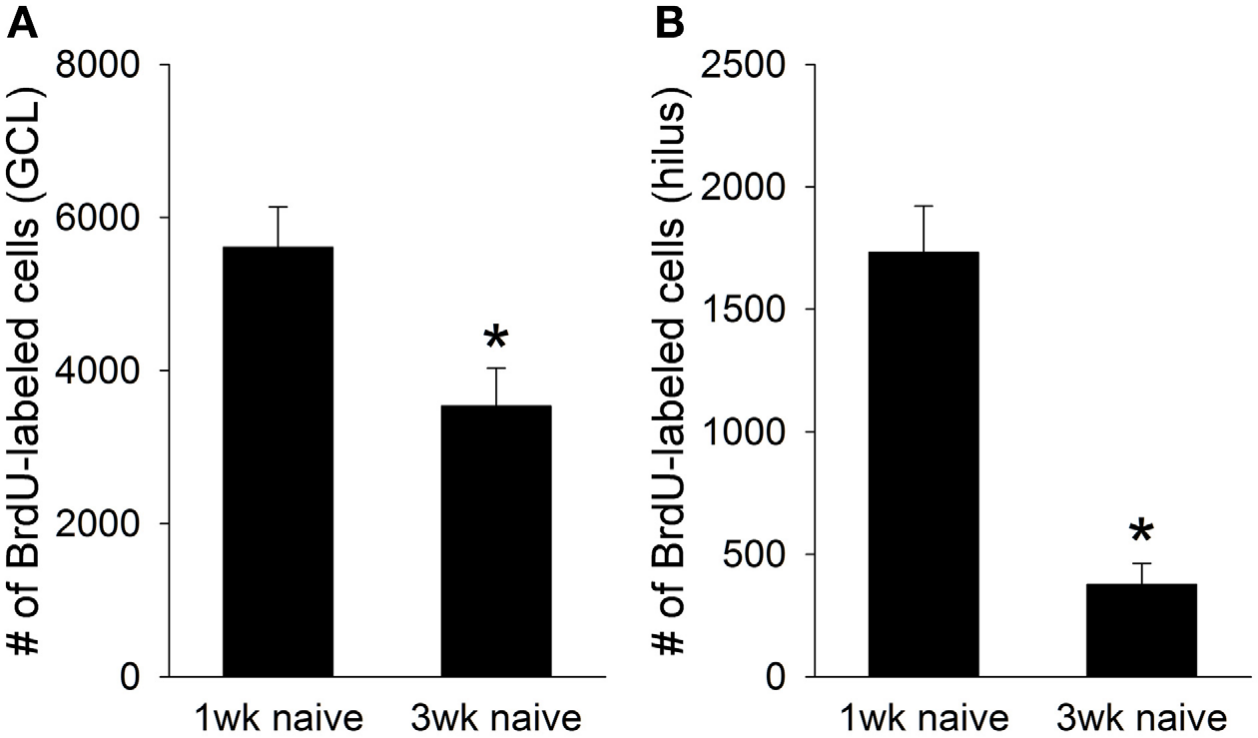
**Naïve animals perfused one week after the BrdU injection (*N* = 6) retained approximately 7,500 BrdU+ cells in the total dentate gyrus**. However, naïve animals perfused three weeks after the BrdU injection (*N* = 10) retained fewer than 4,000 cells, indicating that nearly half of the cells generated in the juvenile dentate gyrus undergo cell death between the first and third week of their birth. This cell death was evident in both **(A)** the granule cell layer (GCL), where nearly 40% of the new cells died, and **(B)** the hilus, where 80% died. Asterisk indicates *p* ≤ 0.05.

Consistent with previous reports, thousands of BrdU+ cells were present in the juvenile dentate gyrus one week after the BrdU injection (Hodes et al., 2009). However, fewer BrdU+ cells were present 3 weeks after the injection [*t*_(14)_ = 3.96, *p* < 0.01]. A large decrease in cell number was observed in both the granule cell layer [GCL; *t*_(14)_ = 2.72, *p* < 0.05; Figure 1A], and the hilus of the dentate gyrus [*t*_(14)_ = 7.43, *p* < 0.01; Figure 1B]. Approximately 40% of the BrdU+ cells in the GCL, and nearly 80% of the BrdU+ cells in the hilus died between the first and third week of their birth. Therefore, a significant number of cells generated in the adolescent dentate gyrus undergo cell death within weeks of their birth, just as they do in the adult dentate gyrus.

**Figure 2:**
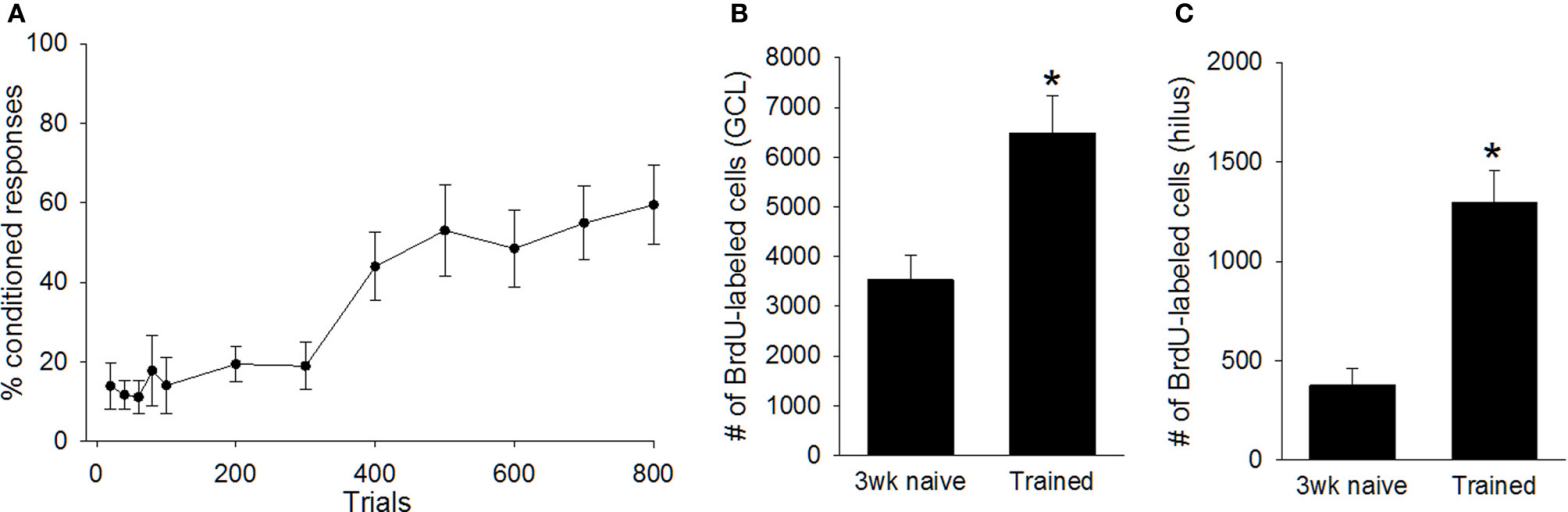
**(A)** As a group, animals trained with trace eyeblink conditioning (*N* = 9) acquired the conditioned response **(B)** Training prevented cell death in the dentate gyrus. Trained animals perfused three weeks after the BrdU injection retained more cells than naive animals perfused three weeks after BrdU injection (*N* = 10). This was observed in both the granule cell layer (GCL), and **(C)** the hilus. Asterisk indicates *p* ≤ 0.05.

**Figure 3:**
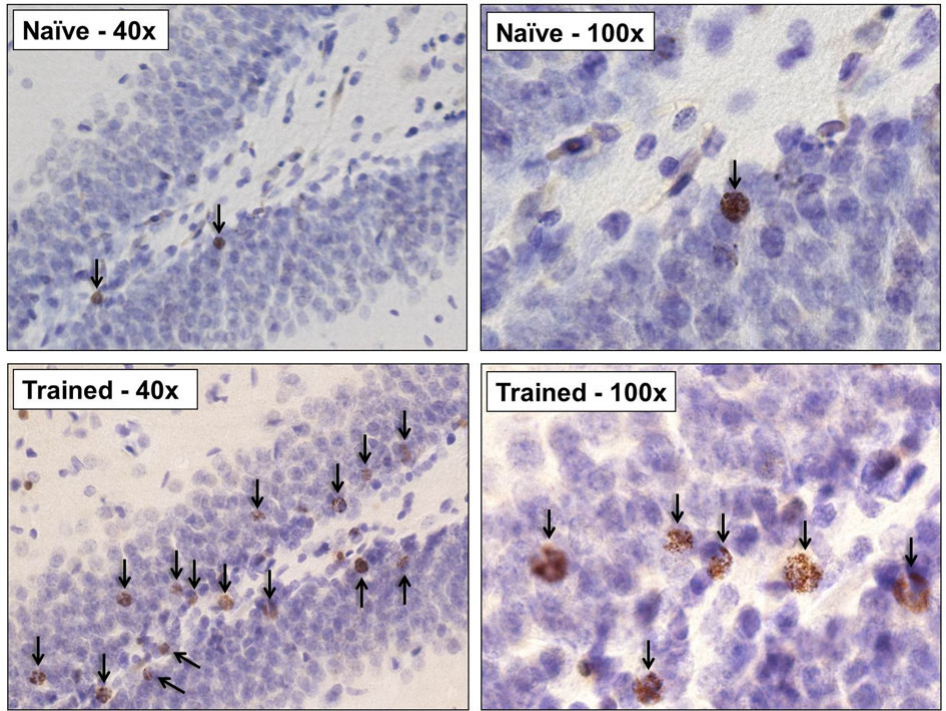
**Representative photomicrographs at 40× and 100× from the dentate gyrus of an untrained animal perfused 3weeks after the BrdU injection, and a trained animal which successfully acquired the trace eyeblink response**. Learning greatly increased the number of surviving BrdU+ cells. Arrows indicate BrdU+ cells.

Next, we determined whether training during puberty prevented the death of cells generated in the juvenile dentate gyrus, as it does during adulthood. Trained animals increased the percentage of CRs emitted across the four sessions (days) of training, as demonstrated with a repeated measures ANOVA [*F*_(7, 56)_ = 9.97, *p* < 0.01; Figure 2A]. The number of cells in trained animals was compared to the number in untrained animals that were sacrificed one, and three, weeks after the BrdU injection. Univariate ANOVA revealed that the number of surviving BrdU+ cells differed between these three groups [*F*_(2, 22)_ = 9.55, *p* < 0.01]. Animals that were trained with trace conditioning retained more new cells than untrained animals that were sacrificed three weeks after the BrdU injection (*p* < 0.01; Figures 2, 3). This difference was observed in both the GCL (*p* < 0.01; Figure 2B), and the hilus (*p* < 0.01; Figure 2C). Moreover, trained animals possessed a similar number of cells as naïve animals that were sacrificed one week after the injection, before the majority of cells underwent cell death.

**Figure 4:**
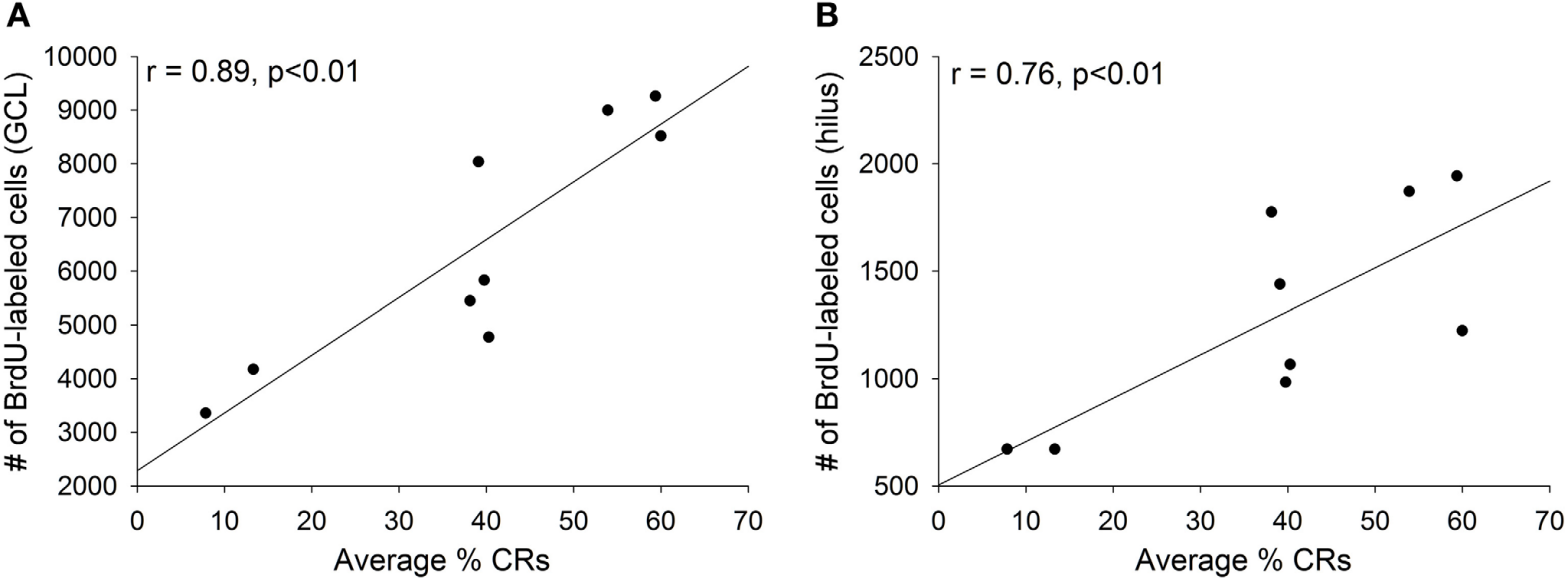
**Animals that successfully acquired the conditioned response retained more new cells than those that failed to learn**. Across all trained animals strong positive correlations were observed between the average percentage of conditioned responses emitted during all four days of training and the number of surviving cells in **(A)** the granule cell layer (GCL) and **(B)** the hilus.

As discussed, training with trace eyeblink conditioning prevents the death of adult-born cells, but only when significant learning occurs. When animals fail to learn, or when they are prevented from learning, training does not increase the number of surviving cells (Waddell and Shors, 2008; Curlik and Shors, 2011). In the current study, we observed strong positive correlations between an individual animal's performance during trace conditioning and the number of surviving BrdU-labeled cells in the animal's dentate gyrus. The average percentage of CRs emitted across all four training sessions strongly correlated with the number of surviving cells in both the granule cell layer (*r* = 0.89, *p* < 0.01; Figure 4A) and the hilus (*r* = 0.76, *p* < 0.01; Figure 4B).

**Figure 5:**
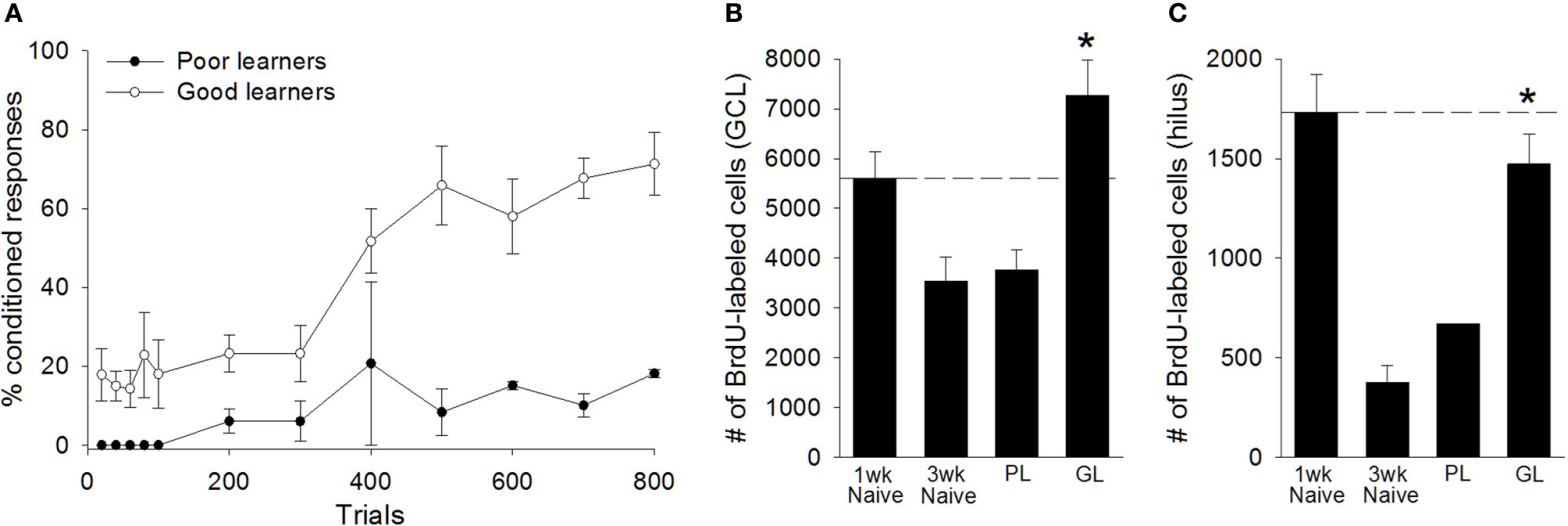
**Learning, not merely training, prevented the death of cells in the peri-pubescent dentate gyrus. (A)** Trained animals were separated into “poor learners” (*N* = 2) and “good learners” (*N* = 7), based on their performance during trace conditioning. Good learners consistently outperformed poor learners. Poor learners, which failed to acquire the conditioned response, retained significantly fewer BrdU+ cells than naïve animals perfused 1week after the BrdU injection (*N* = 6). Moreover, poor learners and naïve animals perfused three weeks after the BrdU injection retained a comparable number of cells, indicating that, in poor learners, the majority of BrdU+ cells underwent cell death between the first and third week of their birth. This cell death was not observed in good learners. Good learners retained significantly more BrdU+ cells than naïve animals perfused three weeks after the BrdU injection. The good learners also retained significantly more BrdU-labeled cells than poor learners. These effects were observed in both **(B)** the granule cell layer (GCL) and **(C)** the hilus of the dentate gyrus. Dashed line corresponds to the number of BrdU+ cells observed in naïve animals one week after the BrdU injection. There was no variance in the number of BrdU+ cells observed in the hilus of poor learners. Asterisk indicates *p* ≤ 0.05.

Based on their performance, we separated animals into “good learners” and “poor learners.” Good learners were those that emitted at least 60% CRs during any block of 100 training trials. Seven trained animals met this criterion, whereas two did not. Repeated measures ANOVA between good learners and poor learners, with training block as the repeated measure, indicated a significant effect of training block [*F*_(7, 49)_ = 4.24, *p* < 0.01], and a significant between groups effect [*F*_(1, 7)_ = 23.47, *p* < 0.01], revealing that good learners outperformed poor learners during training (Figure 5A). Consistent with previous studies of adults, adolescent animals that learned the trace eyeblink response retained significantly more BrdU+ cells in the total dentate gyrus than untrained animals that were sacrificed 3 weeks after the BrdU injection [*t*_(15)_ = 5.06, *p* < 0.01]. This response was evident in both the granule cell layer [*t*_(15)_ = 4.49, *p* < 0.01; Figure 5B] and the hilus [t_(15)_ = 6.79, *p* < 0.01; Figure 5C]. Good learners also retained more BrdU+ cells than animals that failed to learn [*t*_(7)_ = 2.73, *p* < 0.05]. Again, this difference was observed in both the granule cell layer [*t*_(7)_ = 2.52, *p* < 0.05; Figure 5B], and the hilus [*t*_(7)_ = 2.72, *p* < 0.05; Figure 5C]. Animals that failed to learn retained no more BrdU+ cells than untrained animals that were perfused 3 weeks after the BrdU injection. This was observed in the complete dentate gyrus [*t*_(10)_ = 0.39, *p* > 0.05], the GCL [*t*_(10)_ = 0.20, *p* > 0.05], and the hilus [*t*_(10)_ = 1.48, *p* > 0.05]. Together, these results indicate that approximately half of the new cells produced in the adolescent dentate gyrus underwent cell death within the first few weeks of their birth. However, the vast majority of cells that would have undergone cell death instead survived in adolescent animals that engaged in successful learning.

## Discussion

The present data indicate that the process and time course of cell death in the adolescent dentate gyrus closely mirrors that of the adult dentate gyrus, albeit to an exaggerated degree. As in adults, thousands of new cells are produced each day during puberty, but the majority of these cells undergo cell death within 1–3 weeks of their birth (Gould et al., 1999; Epp et al., 2007; Anderson et al., 2011). In both adults and adolescents, much of the cell death can be prevented by engaging in difficult and effortful training procedures, including associative learning of a trace eyeblink response (Dalla et al., 2007; Curlik and Shors, 2011).

During puberty, one injection of BrdU labeled more than 7,000 new cells within a week. However, most of the cells were no longer present 3 weeks after the injection, unless the animals successfully learned the trace eyeblink conditioning procedure. After the learning experience, more than 7,000 cells were observed, suggesting that the cells that are produced in the dentate gyrus during puberty continue to replicate, and can be rescued from death by learning. These results are consistent with those from adult animals, which report that learning prevents the death of nearly all cells generated one week prior to the learning experience (Waddell and Shors, 2008). However, and perhaps most importantly, learning itself is critical. Adolescents that successfully learned to emit CRs during the trace interval retained most of the cells that would have otherwise died, whereas adolescent animals that did not emit learned responses did not retain any more cells than untrained animals. Additionally we observed strong positive correlations (*r* ≈ −0.90) between how well adolescent animals learned, and the number of surviving BrdU+ cells. These results are consistent with those from adults, which have revealed that training increases the number of surviving cells in the adult dentate gyrus, provided that learning occurs, and it is effortful (Curlik and Shors, 2013). Because the adolescent dentate gyrus generates thousands more cells than the adult dentate gyrus (Seki and Arai, 1995; Cameron and McKay, 2001), our results reveal that learning during adolescence can have an even greater impact on the structure of the hippocampal formation than learning during adulthood.

Trace eyeblink conditioning is not the only form of learning that prevents the death of new cells in the hippocampus. Acquisition of spatial memories, as well as learning new physical motor skills, increases the number of cells that survive, provided that the task is sufficiently difficult to learn, and learning occurs (Gould et al., 1999; Epp et al., 2007; Curlik and Shors, 2011; Curlik et al., 2013). For example, acquisition of the hidden platform version of the Morris water maze, or an accelerating rotarod procedure, greatly increases the number of cells that survive in the adult dentate gyrus, whereas acquisition of more easily-acquired skills, such as the visible platform version of the water maze, or a non-accelerating version of the rotarod procedure does not (Gould et al., 1999; Curlik et al., 2013). We did not test these additional forms of learning in the current experiment, but given the similar responses to trace conditioning, it seems likely that effortful acquisition of spatial memories and physical motor skills will likewise increase the number of surviving cells in the adolescent dentate gyrus.

Despite the similarities between adult and adolescent neurogenesis, there are also some striking differences, namely the number of BrdU-labeled cells in the hilus. In adults, we observed very few new cells within the hilus, with no consistent effect of learning on their survival (Gould et al., 1999). However, in this study, many BrdU-labeled cells were present in the hilus, and nearly 80% of them died within weeks. We did not double label these cells, and therefore cannot confirm that they are neurons; however, others have reported an increase in the presence of young neurons during puberty, especially after seizures or alcohol withdrawal (McClain et al., 2013). Again, we did not confirm the neuronal identity of these new hilar cells, but it is nonetheless striking that so many of them were not only generated, but also rescued from death by learning. Animals that successfully learned the CR retained nearly all of the new hilar cells. In the end, 350% more new cells were present in the hilus of pubescent animals that learned, than in animals that did not learn. The functional significance of these presumably “ectopic” granule neurons is unknown. It has been reported that granule neurons in the hilus can extend anatomical connections into CA3, and express similar intrinsic neurophysiological properties to granule cells that reside in the granule cell layer (Scharfman et al., 2007). Therefore, these putatively ectopic granule cells in the adolescent dentate gyrus may function like granule cells in the granule cell layer, but they also may not. It will be important to further characterize the new hilar cells that are apparently so responsive to learning during puberty.

Neurogenesis decreases with chronological age, and the steepness of the decline reportedly depends on species. The number of new cells in a mouse hippocampus can decrease 10-fold from puberty into adulthood, whereas numbers may only decrease 4-fold in humans. It is not yet possible to directly assess the exact number of new cells produced in humans across the lifespan, and therefore these species differences are estimates (Ben Abdallah et al., 2010; Gil-Mohapel et al., 2013; Spalding et al., 2013). With that caveat in mind, there are reported species differences in turnover as well. In rodents, there is apparently less turnover, and therefore more cells are added to the hippocampus over time (Imayoshi et al., 2008); whereas in humans, hippocampal cells are more likely to turnover, and as a consequence fewer cells accrue (Spalding et al., 2013). Some of these changes across the lifespan are presumably mediated by changes in the concentration of reproductive hormones (Galea et al., 2006). For example, testosterone is implicated because its release increases in males during puberty, and decreases with age, along with cell number. However, the effect of reproductive hormones on cell survival is less clear. One study reported that removal of testosterone through gonadectomy decreased the survival of neurons in the adult, but not pubescent, dentate gyrus (Ho et al., 2012). However others report that gonadectomy prior to the onset of puberty increased neuronal survival in the adolescent rhesus macaque (Allen et al., 2014). Together, these results suggest that reproductive hormones likely contribute to the enhanced neurogenesis during puberty, but may not fully explain the enhanced neurogenic response to learning that we report here.

New neurons in the adult hippocampus are implicated in a number of learning processes, including trace eyeblink conditioning, trace fear conditioning, cognitive flexibility, and discrimination learning (Shors et al., 2001, 2002, 2012; Achanta et al., 2009; Sahay et al., 2011; Burghardt et al., 2012). Others report that the new cells may play a role in processes more related to memory (Gu et al., 2012). Still others report correlations between the numbers of cells generated and learning ability (for exception see Anderson et al., 2011). Of course, there are correlations among learning, age and neurogenesis, as aged animals produce fewer cells than young animals, and are often impaired in tests of learning and/or memory. For example, the number of new neurons in the hippocampus correlates significantly with spatial performance during water maze training across the lifespan (Gil-Mohapel et al., 2013). Specifically, very young animals with high levels of neurogenesis were more likely to use efficient search strategies during water maze training, whereas aged animals with very low levels of neurogenesis were more likely to use indirect, or random, search strategies. This does not mean that new cells are necessary for this type of learning, and indeed many studies indicate that they are not (Shors et al., 2002). That said, it remains possible that the process of neurogenesis has a disproportionate effect on learning during this early developmental period, perhaps one that is more critical for learning to occur than during adulthood. For example, it has been reported that disrupting neurogenesis preferentially impaired spatial learning in juvenile, but not in adult, mice (Martinez-Canabal et al., 2013). Similarly, disrupting neurogenesis altered social behavior in juvenile, but not adult, animals (Wei et al., 2011). Together, these results suggest that disrupting neurogenesis during adolescence imposes a greater risk to mental development, perhaps because so many more cells are produced. Such a conclusion could have meaningful implications for humans. For example, chemotherapy typically delivers antimitotic drugs into the blood, which dramatically reduce the number of new neurons produced in the hippocampus, and result in cognitive impairments, often referred to as chemobrain (Nokia et al., 2012a). Given the data presented here and elsewhere, treatment with antimitotics in children and young adults may impose an even greater impact on brain structure and function than similar treatment in adults.

In the end, it is important to consider why the pubescent animal produces so many new cells, in the hippocampus and elsewhere, and why cells in the pubescent animal are especially responsive to learning. On one hand, it seems obvious why they would do so, given the numerous new experiences and challenges that arise as a young animal leaves the relative safety and comfort of its mother. However, it is not readily apparent how new neurons function to enhance survival of the individual. In previous work, we proposed that the production and rescue of new neurons by learning enhances learning in the future which thereby allows even more new neurons to survive (Shors, 2008; Shors et al., 2012). In one study, adult animals were trained to learn a new skill that rescued new neurons from death. Learning the skill enhanced learning of a similar skill, which on its own also increases cell survival. As a result, cells that were not even present at the time of the first learning experience were more likely to survive as a result of the future learning abilities (Nokia et al., 2012b). We would submit that this process, referred to as “learning to learn,” is especially engaged during early development to ensure that learning occurs rapidly and successfully with structural, and indeed permanent, consequences. These processes interact with one another to produce a positive feedback system, which enhances the survival not only of neurons, but of individuals.

### Conflict of interest statement

The authors declare that the research was conducted in the absence of any commercial or financial relationships that could be construed as a potential conflict of interest.
